# Level and significance of plasma myeloperoxidase and the neutrophil to lymphocyte ratio in patients with coronary artery disease

**DOI:** 10.3892/etm.2014.2034

**Published:** 2014-10-17

**Authors:** FADIA A. MAYYAS, MOHAMMAD I. AL-JARRAH, KHALID S. IBRAHIM, KAREM H. ALZOUBI

**Affiliations:** 1 Department of Clinical Pharmacy, Faculty of Pharmacy, Jordan University of Science and Technology, Irbid 22110, Jordan; 2 Department of General Medicine, Faculty of Medicine, Jordan University of Science and Technology, Irbid 22110, Jordan; 3 Princess Muna Heart Institute, King Abdullah University Hospital, Irbid 22110, Jordan; 4 Department of General Surgery, Division of Cardiovascular Surgery, Faculty of Medicine, Jordan University of Science and Technology, Irbid 22110, Jordan

**Keywords:** myeloperoxidase, neutrophil, myocardial infarction, coronary artery disease

## Abstract

Inflammation plays a pivotal role in the etiology of coronary artery disease (CAD). Myeloperoxidase (MPO) is a potent inflammatory factor and a critical modulator of coronary inflammation and oxidative stress. The goal of this study was to determine the impact of the plasma MPO (pMPO) level and neutrophil/lymphocyte ratio on the clinical characteristics and outcomes of patients with CAD. Blood samples were collected from 210 patients with underlying chest pain or recent myocardial infarction (MI) prior to coronary angiography in order to measure pMPO levels. The pMPO levels and neutrophil/lymphocyte ratio were correlated with clinical characteristics and outcomes following catheterization. The pMPO level and neutrophil/lymphocyte ratio were higher in patients with recent MI than in patients with CAD (coronary occlusion ≥50%) or without CAD (coronary occlusion <50%). Patients with ST segment elevated MI (STEMI) had a higher neutrophil/lymphocyte ratio relative to patients with non-STEMI. The pMPO level was identified to correlate with the neutrophil/lymphocyte ratio and the need for coronary artery reperfusion by coronary artery bypass surgery or percutaneous coronary intervention. Patients who were taking aspirin had lower pMPO levels and neutrophil/lymphocyte ratio compared with those who were not taking aspirin. The plasma neutrophil/lymphocyte ratio was negatively associated with the left ventricular ejection fraction at baseline and the 30-day follow-up, whereas pMPO showed no correlation. Multivariate analysis indicated that the pMPO level was positively associated with MI, the neutrophil/lymphocyte ratio and coronary intervention. The preoperative use of aspirin was associated with a lower pMPO level and neutrophil/lymphocyte ratio. In conclusion, pMPO is positively associated with MI, the neutrophil/lymphocyte ratio and coronary intervention. The preoperative use of aspirin is associated with a lower pMPO level and neutrophil/lymphocyte ratio. pMPO may serve as a predictor of coronary intervention and as a potential therapeutic target for the reduction of inflammation in patients with CAD.

## Introduction

The principal mechanisms underlying the development of coronary artery atherosclerosis and myocardial infarction (MI) are numerous and complex. Coronary artery disease (CAD) is associated with significant patient morbidity and mortality. A larger infarction size and greater mortality are associated with ST-segment elevation myocardial infarction (STEMI) compared with non-STEMI (1).

Inflammation is increasingly being considered as a key player and critical feature in coronary atherosclerosis (2). Previous studies have observed that areas of plaque rupture are associated with increased levels of inflammatory cells (3,4). In addition, coronary plaques have been observed to be infiltrated by diffused inflammatory cells, including neutrophils, in patients who succumbed following MI (3,5). The neutrophil/lymphocyte ratio is known to be an important predictor of adverse outcomes in MI (6).

Factors that promote endothelial dysfunction, inflammatory reactions and platelet aggregation may result in vascular dysfunction and atherosclerosis. Plasma levels of inflammatory biomarkers such as C-reactive protein (CRP) have been shown to increase and predict atherothrombotic events (7) suggesting that inflammation contributes to MI (2).

Neutrophils are an important source of the myeloperoxidase enzyme (MPO), a peroxidase and an inflammatory factor. MPO is present in atherosclerotic plaques and may aggravate cardiac ischemia (8). MPO has been shown to promote the oxidation of low density protein (LDL) (9) and apolipoprotein A-I (10), thus, reducing its ability to cause cholesterol efflux. Additionally, MPO promotes endothelial dysfunction (11) and apoptosis/detachment, promoting plaque rupture (2,12). Thus, MPO may mediate the development of atherosclerotic plaques in patients with CAD and may increase the risk of acute coronary syndrome (ACS) (13). However, it is unclear whether the plasma MPO (pMPO) level predicts the severity of CAD and the coronary intervention required for the reperfusion of atherosclerotic arteries.

The goal of the present study was to assess the correlation of the pMPO level and neutrophil/lymphocyte ratio with clinical characteristics, the need for coronary reperfusion and the left ventricular ejection fraction (LVEF) prior to coronary angiography in patients with CAD.

## Methods

### Patient selection

Inclusion criteria of this study were patients who presented to King Abdullah University Hospital (KAUH; Irbid, Jordan) with chest pain (angina) or recent MI (presented within less than a week of the onset of symptoms). The majority of the patients were referred from peripheral hospitals to KAUH and underwent coronary angiograms for diagnosis and/or reperfusion when required by percutaneous coronary intervention (PCI) or coronary artery bypass graft surgery (CABG).

All included patients provided written informed consent, and the study was conducted with approval from the Institutional Review Board of KAUH. Demographic/clinical characteristics and laboratory tests were obtained prospectively from patients and from their medical records. Plasma lipids levels, inflammatory cell percentages (neutrophils, lymphocytes and monocytes), and glucose levels were obtained from patients’ data registry at KAUH. Data concerning ejection fraction, left atrial (LA) size, need for revascularization, and the use of medication prior to hospitalization were also collected at baseline (for 210 patients) and at 30 days follow-up (for 100 patients). A detailed clinical history, physical examination and relevant laboratory investigations were used to confirm the presence of CAD or MI.

Levels of the cardiac biomarkers troponin I, troponin T, creatine kinase (CK) and/or CK-MB, were measured to document MI. The diagnosis of MI was established according to World Health Organization (14) and American Heart Association/American College of Cardiology criteria (1), with a history of chest pain lasting >20 min, the presence of cardiac enzyme leakage and characteristic electrocardiogram (ECG) changes. The presence of an elevated ST segment/Q wave on the ECG indicates STEMI. Angina was defined as chest pain at rest or during exertion with a slight or significant limitation of daily physical activity. Exclusion criteria were patients with recent infection, patients with heart failure (HF) without evidence of CAD, and patients who had suffered an MI attack within the month prior to their current chest pain or MI.

### Echocardiographic and coronary angiographic analysis

Echocardiographic studies were conducted using a two dimensional imaging system [ALT (model HD1) 6,000 ht with a 2–4 MHz probe; Philips Medical Systems, Inc., Bothell, WA, USA] to evaluate the LA diameter (size) and LVEF. The LA size was indexed to the body surface area. Coronary catheterization was used to confirm the presence of CAD (coronary occlusion ≥50%) in one or more of the main coronary arteries.

### Blood collection

Blood was collected from the femoral artery in EDTA tubes during femoral artery puncture prior to catheterization and before the administration of adjunctive drug therapy for all patients. Samples were brought to the laboratory immediately and centrifuged at 700 × g for 15 min to separate the plasma. Samples were stored at −80°C until the analysis of pMPO.

### pMPO measurements

pMPO concentrations prior to catheterization were determined using instant enzyme-linked immunoassays (BMS2038INST; eBioscience, Inc., Vienna, Austria). Human plasma samples (diluted 1:50), controls and standards were pipetted in wells coated with biotin-conjugated mouse anti-human MPO monoclonal antibody bound to streptavidin-horseradish peroxidase and incubated on a horizontal shaker at 400 rpm at room temperature. Standards and samples were assayed in duplicate. Following 3 h of incubation, the contents of the wells were washed, and then 100 μl tetramethylbenzidine (TMB) substrate was added for 10 min followed by 100 μl stop solution. Absorbance values were immediately determined using an ELISA reader (EL×800, Bio-tek instruments, Winooski, VT, USA) at 450 nm, using 620 nm absorbance as a reference. The quantity of MPO was interpolated from a calibration curve of standards. The assay was sensitive (lower limit of detection, 0.026 ng/ml), specific and reproducible with calculated overall inter- and intra-assay coefficients of variation of <10 and <6%; respectively.

### Plasma inflammatory cell measurements

Plasma levels of white blood cells (WBCs) and percentages of neutrophils, lymphocytes and monocytes were measured using a Beckman Coulter (LH 780) Hematology Analyzer (Coulter Technology Center, Miami, FL, USA).

### Statistical analysis

Continuous variables are presented as mean ± standard error of the mean (SEM) while categorical data are presented as numbers and percentages. Univariate and multivariate analyses were used to evaluate the association of demographic and clinical parameters with pMPO levels and the neutrophil/lymphocyte ratio. Normally distributed variables were analyzed using analysis of variance or t-test. Non-normally distributed variables were analyzed using Mann-Whitney U test and Kruskal-Wallis test. To compare frequencies among study groups, the Chi-square test was used. Spearman’s correlation was used to assess the correlation between variables. Univariate analysis was performed and figures created using GraphPad Prism 5 (GraphPad Software, Inc., La Jolla, CA, USA). pMPO levels were not normally distributed; thus, a log transformation of MPO levels was used in the multivariate analysis to normalize the dataset. Multivariate analyses were performed using JMP11 software (SAS Institute Inc., Cary, NC, USA). P<0.05 was considered to indicate a statistically significant difference.

## Results

### Patient characteristics

Plasma samples from 210 patients were used for the biochemical analysis of MPO protein levels and inflammatory cell measurements. Study groups were stratified based on their clinical presentation, coronary angiographic findings and cardiac enzyme levels. The study groups comprised: i) patients who presented with negative cardiac enzymes and normal main coronary arteries with <50% stenosis (CAD <50% group, n=60), ii) patients with negative cardiac enzymes and CAD with ≥50% stenosis in one or more of the main coronary arteries (CAD ≥50% group, n=68); and iii) patients that presented with recent MI (within the last week) as documented by cardiac enzyme leakage and coronary angiogram (MI group, n=82). The MI group was further subdivided into two groups based on ECG findings: STEMI (n=41) and non-STEMI (n=41). Certain STEMI patients (n=27) had received thrombolytic agents prior to referral to KAUH. Patients with recent MI underwent emergency catheterization (within <24 h, 42%) or elective catheterization (within 24 h to 1 week, 57%). A total of 11 patients were scheduled for CABG. Hypertension (HT) and diabetes mellitus (DM) were present in 67.9 and 42.4% of patients, respectively (Table I). There were 25 patients that presented with heart failure (HF), seven of whom had CAD and 18 of whom had MI. The majority of patients with MI presented with acute HF (n=15) whereas three patients with MI and seven patients with CAD presented with a history of chronic HF.

### Univariate predictors of pMPO and inflammatory cell levels

pMPO and inflammatory cell levels were evaluated as factors in the clinical manifestation of CAD (CAD status graded as 0, CAD with <50% stenosis; 1, CAD ≥50%; and 2, MI) and the clinical outcome.

**Figure 1:**
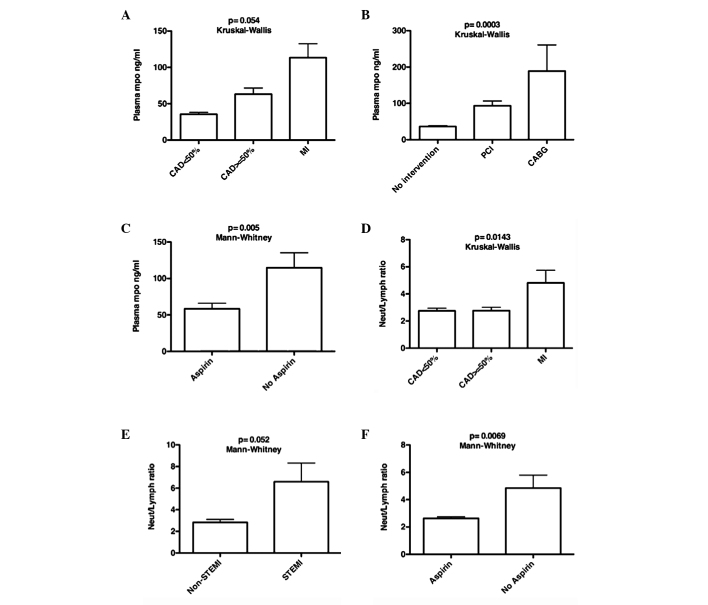
Plasma levels of MPO and the neutrophil/lymphocyte (neut/lymph) ratio in patients with coronary artery disease (CAD). (A) Plasma levels of MPO in patients with CAD <50%, CAD ≥50%, and recent MI. (B) Plasma levels of MPO among patients who underwent CABG, PCI or no intervention. (C) Plasma levels of MPO in patients who were/were not on aspirin prior to catheterization. (D) Plasma neut/lymph ratio in patients with CAD <50%, CAD ≥50%, and recent MI. (E) Plasma neut/lymph ratio in patients with ST segment elevated MI (STEMI) and non-STEMI. (F) Plasma neut/lymph ratio in patients who were/were not on aspirin prior to catheterization. MPO, myeloperoxidase; MI, myocardial infarction; CABG, coronary artery bypass graft; PCI, percutaneous coronary intervention.

The levels of pMPO in patients with recent MI were higher than those in patients with CAD ≥50% and those with <50% coronary artery stenosis (mean ± SEM, 113.39±19.16, 63.134±8.40 and 35.61.±2.62 ng/ml, respectively; P=0.054, Kruskal-Wallis test; Fig. 1A). The pMPO levels were not statistically different between the STEMI and non-STEMI groups (146.878±33.98 vs. 79.905±16.64 ng/ml; P=0.696, Mann-Whitney test). The pMPO levels also did not differ in patients with STEMI according to whether they received or did not receive thrombolytic agents, or among those who underwent elective or emergency catheterization (P<0.05). pMPO levels were higher in patients who were scheduled for CABG compared to those in patients who underwent PCI or no intervention (189.01±71.76, 93.75±12.96, 36.20±2.31 ng/ml, respectively; P=0.0003, Kruskal-Wallis test; Fig. 1B). pMPO levels in patients who took aspirin prior to hospitalization were lower compared with those in patients who did not take aspirin (58.47±7.70 vs. 114.76±20.52 ng/ml; P=0.005, Mann-Whitney test; Fig. 1C).

The white blood cell count (WBC) and neutrophil percentage were higher in the MI group compared with those in the CAD <50% and CAD ≥50% groups (Table I). The neutrophil/lymphocyte ratio also correlated with CAD status (4.75±0.93, 2.61±0.17 and 2.72±0.19 in the MI, CAD≥50% and CAD<50% groups, respectively; P=0.0143, Kruskal-Wallis test; Fig. 1D). The neutrophil/lymphocyte ratio was higher in patients with STEMI than in patients with non-STEMI (6.59±1.73 vs. 2.83±0.27, respectively; P=0.052, Mann-Whitney test; Fig. 1E) and tended to be higher in patients who were scheduled for CABG relative to patients who required PCI or no intervention (5.33±1.42, 3.46±0.54 and 2.72±0.15; respectively; P=0.07, Kruskal-Wallis test). Patients who were taking aspirin had a lower plasma neutrophil percentage relative to those who were not taking aspirin (63.02±0.82 vs. 67.66±1.84%; P=0.047, Mann-Whitney test) and had a lower neutrophil/lymphocyte ratio (2.62±0.12 vs. 4.88±0.96; P=0.0069, Mann-Whitney test; Fig. 1F).

The pMPO level was not identified to be associated with HF (regardless of chronic or acute presentation), hypertension, diabetes, smoking, indexed LA size, use of statins prior to admission to hospital, angiotensin converting enzyme inhibitors (ACEi)/receptor blockers (ARBs), or β blockers. However, the pMPO level was found to be positively correlated with the neutrophil percentage (Spearman’s r=0.207, P=0.0115) and the neutrophil/lymphocyte ratio (r=0.253, P=0.0019), but not with the WBC count (P=0.226) or monocyte percentage (P=0.523). In addition, the pMPO level did not correlate with the baseline LVEF (P=0.212), or the levels of high density lipoprotein (HDL; P=0.35), LDL (P=0.588) or total cholesterol (P=0.148). However, plasma inflammatory cells were negatively correlated with the baseline LVEF; WBC (r=−0.145, P=0.073), the neutrophil percentage (r=−0.204, P=0.027), the neutrophil/lymphocyte ratio (r=−0.264, P=0.004) and monocyte percentage (r=−0.227, P=0.0137). The plasma neutrophil/lymphocyte ratio correlated with HF (2.94±0.18 vs. 6.13±2.23; P=0.044, Mann-Whitney test) and tended to be higher in patients with acute HF (8.39±4.08).

### Independent predictors of pMPO level

Multivariate analysis was performed to determine independent predictors of pMPO level. By step-wise analysis, it was identified that diabetes, HF, LVEF, HT, smoking, plasma lipids, and the use of ACEi/ARBs, statins or β blockers did not correlate with pMPO level; thus, they were excluded from the final model. By step-wise analysis adjusting for age, BMI and gender, pMPO was found to be associated with CAD status (graded as 0, CAD <50% stenosis; 1, CAD ≥50% stenosis; 2, MI; P=0.008). As the CAD status was collinear with coronary intervention (graded as 0, no intervention; 1, PCI; 2, CABG), it was not presented in the final model to avoid masking the correlation of pMPO with coronary intervention. Adjusting for age, gender and BMI, multivariate analysis showed that the neutrophil/lymphocyte ratio and coronary intervention were significantly and positively associated with the pMPO level (Table II). Notably, this analysis revealed that patients who took aspirin had lower pMPO levels than patients who did not take aspirin (P=0.0155).

### Independent predictors of the plasma neutrophil/lymphocyte ratio

Multivariate analysis was used to evaluate independent predictors of the plasma neutrophil/lymphocyte ratio. By step-wise analysis, it was identified that diabetes, HT, smoking, plasma lipids, and the use of ACEi/ARBs, β blockers or statins did not correlate with the neutrophil/lymphocyte ratio; thus, they were excluded from the model. By step-wise analysis adjusting for age, BMI, and gender, the neutrophil/lymphocyte ratio was found to be associated with CAD status (P=0.018) and coronary intervention (P=0.05). As both CAD status and coronary intervention were collinear with LVEF, they were excluded from the final model to avoid masking the correlation of LVEF with the neutrophil/lymphocyte ratio. Adjusting for age, gender and BMI, multivariate analysis showed that both baseline LVEF and the use of aspirin were negatively correlated with the neutrophil/lymphocyte ratio (Table III). Similarly, HF was also independently associated with the neutrophil/lymphocyte ratio (P<0.0001), but was not presented in the model due to it being collinear with LVEF.

### Prognostic value of the baseline pMPO level and the neutrophil/lymphocyte ratio at the 30 day follow-up

A total of 100 patients (41 from the MI group, 34 from the CAD ≥50% group and 25 from the CAD <50% group) were followed up for clinical assessment 30 days after angiography. Two patients required coronary reperfusion; one presented with recurrent MI (pMPO, 12.71 ng/ml) and one presented with restenosis requiring PCI (pMPO, 21.19 ng/ml). One patient who presented with recurrent angina underwent angiography without need for stenting (pMPO, 293.19 ng/ml). By multivariate analysis adjusting for age, gender, BMI and smoking, the baseline pMPO was not associated with LVEF at 30 days or with the change in LVEF at the 30-day follow-up (P>0.05; data not shown). The plasma neutrophil/lymphocyte ratio was correlated negatively with LVEF after 30 days (Spearman’s r=−0.402, P=0.0007), but was not associated with the change in LVEF after 30 days (P=0.251). By this time, the increase in LVEF was minimal and not significant (mean % increase in LVEF: all patients, 3.11%±1.34, P=0.53; CAD <50%, 1.28±2.22; CAD ≥50%, 1.22±2.57; and MI, 5.55±2.10; P=0.18, Kruskal-Wallis).

Adjusting for age, BMI, gender and use of aspirin, the pMPO and neutrophil/lymphocyte ratio were not found to be associated with the change in LVEF at the 30-day follow-up (P=0.240 and P=0.812, respectively; data not shown).

## Discussion

MPO, an enzyme derived from activated neutrophils and monocytes, is involved in the generation of reactive oxygen species (ROS) and nitric oxide-derived oxidants (8). Thus, MPO promotes inflammation and oxidative stress and contributes to endothelial dysfunction and plaque formation, rupture and ventricular remodeling following MI (3,12).

The levels and activity of pMPO have been reported to increase in MI patients (15,16) and to predict endothelial dysfunction (11), the risk of ACS (13,17) and mortality (15). In addition, high pMPO levels have been shown to predict 30-day mortality and major adverse clinical events in patients with STEMI (18–20) and long-term adverse clinical events in stable cardiac patients (19). However, it is not known whether this relationship is similar for other clinical presentations of CAD. In the present study, it was found that pMPO levels were elevated in the patients with recent MI compared with those in patients with or without CAD and correlate with the neutrophil/lymphocyte ratio, suggesting that the increased circulatory neutrophils promote MPO release, plaque formation and the development of MI. Neutrophil infiltration is actively associated with plaque rupture and MI (3). The neutrophil/lymphocyte ratio is known to be as strong predictor of adverse outcomes in patients with STEMI (6). Notably, in the present study, the neutrophil/lymphocyte ratio was correlated with CAD status and was higher in STEMI than non-STEMI. As MPO is derived from activated neutrophils and monocytes, a higher pMPO level in patients with STEMI relative to those with non-STEMI was expected; however, the differences in data did not reach statistical significance.

The current study provides important insights regarding the pathophysiologic significance of pMPO in patients with CAD. One important finding is that the pMPO level is higher in patients undergoing CABG than in those undergoing PCI or no intervention. Similarly, the neutrophil/lymphocyte ratio was found to correlate with the type of coronary intervention, suggesting that plasma inflammatory neutrophils and MPO contribute significantly to the extent and severity of CAD and could be used clinically to predict the appropriate choice of clinical intervention required for the reperfusion of atherosclerotic arteries.

Aspirin is a commonly prescribed anti-platelet non-steroidal anti-inflammatory drug (NSAID) for preventing ACS and mortality in CAD patients. The effects of NSAIDs occur through the inhibition of prostaglandin H synthase, which is produced from arachidonic acid via the actions of cyclooxygenase and peroxidase (21). In the present study, the use of aspirin was identified to be associated with a reduced neutrophil/lymphocyte ratio and lower levels of pMPO, which might further explain the use of aspirin and its benefits in the prevention of MI and mortality. Similarly, the use of aspirin has previously been shown to reduce MPO activity (22) and neutrophil levels (23).

LVEF is an important determinant of cardiac function and a predictor of morbidity and mortality (24). In the present study, the pMPO levels were not correlated with baseline LVEF, 30-day follow-up LVEF or the change in LVEF during follow-up. However, inflammatory cell percentages, including the neutrophil/lymphocyte ratio, were significantly correlated with HF, and with baseline and 30-day LVEF. A previous study identified a correlation between LVEF and WBC count (18), clearly indicating that inflammation is significant in LV dysfunction and the development of HF. The lack of a correlation between pMPO and LVEF suggests the presence of other inflammatory mediators that may contribute more significantly to LVEF. The changes in LVEF at 30 days were not significant, which might also explain the lack of correlations of the pMPO level and neutrophil/lymphocyte ratio with changes in LVEF after 30 days.

The present study demonstrates an association of pMPO with MI in relation to patients with and without CAD who have a low risk of ACS compared with other groups.

However, the present study does not provide information regarding the kinetics of the increase and decrease of pMPO levels. One limitation of the study is the inclusion of patients with MI who presented within a week and received drug therapies that might have affected the pMPO levels. However, pMPO levels were not different between patients who received thrombolytics relative and those who did not receive them. Similarly, pMPO levels have been shown to be similar in patients with MI regardless the use of thrombolytic therapy (25).

MPO is a key inflammatory mediator associated with MI, the neutrophil/lymphocyte ratio and the type of coronary intervention, suggesting that it may contribute to inflammation and the increased risk, severity, and extent of CAD in patients. The association of the neutrophil/lymphocyte ratio with low ejection fraction further suggests that inflammation promotes the development of ventricular dysfunction and HF in patients with CAD. The use of interventions that reduce pMPO production, such as aspirin, limit MI development and related adverse outcomes. This study suggests the use of pMPO levels and the neutrophil/lymphocyte ratio as CAD risk markers and clinical predictors for reperfusion by PCI or CABG. It may also help with the selection of patients who may benefit from use of additional treatments that reduce pMPO and neutrophil levels.

## Figures and Tables

**Table I:** Patients characteristics.

Variables	CAD <50% (n=60)	CAD ≥50% (n=68)	MI (n=82)	P-value
Clinical characteristics				
Age, years	53.7±1.44	60.7±1.38	56.1±1.17	0.0012^a^
Male gender, n (%)	27 (45.0)	46 (67.6)	68 (82.9)	<0.0001^a^
Body mass index	30.33±0.87	28.88±0.70	28.46±0.51	0.1440
Hypertension, n (%)	46 (77.9)	53 (77.9)	43 (52.4)	0.0006^a^
Heart failure, n (%)	0 (0)	7 (10.2)	18 (21.9)	0.0003^a^
Diabetes mellitus, n (%)	22 (36.6)	35 (53.0)	30 (37.9)	0.1057
Current smoking, n (%)	20 (33.9)	25 (36.7)	47 (57.3)	0.0075^a^
Laboratory tests				
White blood cell count	8.23±0.30	7.77±0.29	9.77±0.38	0.0003^a^
Neutrophils %	63.55±1.25	62.33±1.17	68.00±1.68	0.0486^a^
Monocytes %	6.59±0.26	8.54±1.54	7.94±0.45	0.0921
Neutrophils/lymphocytes	2.72±0.19	2.61±0.17	4.75±0.93	0.0143^a^
Low density lipoprotein, mmol/l	3.29±0.18	2.81±0.15	3.17±0.13	0.0655
High density lipoprotein, mmol/l	1.08±0.04	1.00±0.04	0.91±0.02	0.0112^a^
Triglycerides, mmol/l	2.19±0.15	2.44±0.21	2.35±0.17	0.7831
Total cholesterol, mmol/l	4.95±0.20	4.39±0.15	4.73±0.14	0.0669
Fasting plasma glucose	7.44±0.54	10.67±0.92	8.24±0.64	0.0145^a^
HBA1C	6.83±0.32	7.41±0.34	7.05±0.36	0.5085
Intervention, n (%)				
PCI	0 (0)	47 (69.1)	65 (79.2)	<0.0001^a^
Coronary artery bypass graft	0 (0)	4 (5.8)	7 (8.6)	0.0820
Pre-operative echocardiographic data				
Indexed LA diameter, cm/m^2^	2.04±0.05	2.09±0.06	2.01±0.03	0.8892
Left ventricular ejection fraction	56.45±0.93	50.57±1.41	46.60±1.39	<0.0001^a^
Pre-operative use of medication, n (%)				
ACE inhibitors	22 (36.6)	20 (29.8)	17 (20.7)	0.1070
ARBs	12 (20.0)	13 (19.4)	6 (7.3)	0.0487^a^
Diuretics	14 (23.3)	16 (23.8)	9 (10.9)	0.0722
β blockers	37 (61.6)	40 (59.7)	33 (40.2)	0.0153^a^
Statins	33 (55.0)	47 (70.1)	33 (40.2)	0.0011^a^
Aspirin	51 (86.4)	56 (82.3)	40 (48.7)	<0.0001

Values are the mean ± standard error of the mean, unless otherwise indicated. CAD, coronary artery disease; MI, myocardial infarction; HBA1C, glycosylated hemoglobin; LA, left atria; ACE, angiotensin converting enzyme; ARBs, angiotensin-II receptor blockers.

a P<0.05.

**Table II:** Multivariate factors associated with plasma MPO content.

	Response = log plasma MPO ng/ml			
	
Variable	Estimate (slope, B)	Standard error	P-value	Standardized coefficient (β)
Age, years	0.0004	0.0060	0.9417	0.07
Female gender	−0.0825	0.1517	0.5875	−0.54
BMI	0.0004	0.0115	0.6849	0.41
Neutrophil/lymphocyte ratio	0.0866	0.0202	<0.0001^a^	4.27
Coronary intervention	0.1561	0.0676	0.0224^a^	2.31
Use of aspirin	−0.3359	0.1652	0.0439^a^	−2.03

BMI, body mass index, Coronary intervention: 0, no intervention; 1, percutaneous coronary intervention; 2, coronary artery bypass surgery. β is the standardized coefficient (slope/standard error).

a P<0.05.

**Table III:** Multivariate factors associated with plasma neutrophils/lymphocytes ratio.

	Response = neutrophil/lymphocyte ratio			
	
Variable	Estimate (slope, B)	Standard error	P-value	Standardized coefficient (β)
Age, years	−0.0166	0.0304	0.5639	−0.55
Female gender	−0.1278	0.7583	0.8666	−0.17
BMI	0.0331	0.0615	0.5916	0.54
Baseline LVEF	−0.1065	0.0349	0.0029^a^	−3.05
Use of aspirin	−1.8282	0.7644	0.0185^a^	−2.39

BMI, body mass index; LVEF, left ventricular ejection fraction. β is the standardized coefficient (slope/standard error).

a P<0.05.
